# Strategies to enhance greenhouse strawberry yield through honeybee pollination behavior: a simulation study

**DOI:** 10.3389/fpls.2024.1514372

**Published:** 2024-12-05

**Authors:** Zhihao Cao, Shuo Jiang, Hongchun Qu

**Affiliations:** ^1^ College of Information Science and Engineering, Zaozhuang University, Zaozhuang, China; ^2^ College of Automation, Chongqing University of Posts and Telecommunications, Chongqing, China

**Keywords:** strawberry, cross-pollination, interplanting, greenhouse cultivation, computer simulation, honeybees

## Abstract

Strawberries are a widely cultivated greenhouse crop in China, primarily pollinated by honeybees, in accordance with traditional planting practices and local conditions. Extensive research has demonstrated that cross-pollination benefits numerous strawberry cultivars, leading to enhanced yield through the interplanting of different cultivars. However, the high costs associated with cultivation have hindered systematic research on the design of interplanting strategies. In this study, we utilized a simulation model to investigate how to leverage honeybee natural foraging behavior to improve pollination efficiency and explore fruit weight under various interplanting strategies within a greenhouse. Our findings indicate that adopting an alternating planting approach for different cultivars within the same bed effectively facilitates cross-pollination, leading to increased strawberry fruit yield. Additionally, dividing the strawberry plants into two batches and staggering their planting time helps mitigating the pressure of competition for bee pollination during peak blooming period, consequently contributing to enhanced yield. These proposed planting strategies offer valuable cultivation suggestions for farmers in some remote areas in China who still rely on honeybees as primary pollinators.

## 1 Introduction

**Figure 1:**
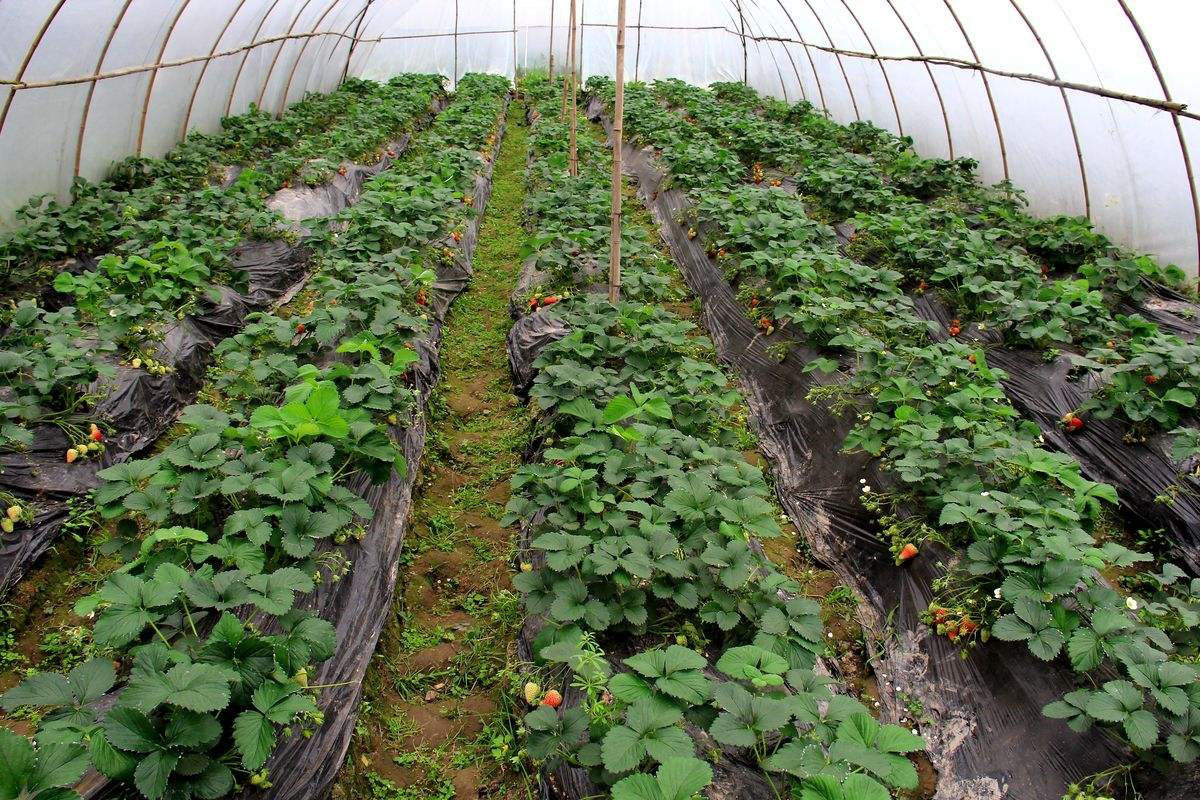
A typical strawberry greenhouse in China.

China is recognized as the world’s largest producer of strawberries (*Fragaria × ananassa Duch*.), with a production of 3,336,690 tons in 2023, maintaining the top position since 1994 according to FAO data (Lei et al., 2021). In China, strawberry cultivation is predominantly conducted in greenhouses (**Figure 1**), a practice driven by the fruit’s fragility and specific climatic requirements, with bees serving as the principal pollinators (Chandler et al., 2012; Barahona-Segovia et al., 2023). Bee pollination, as opposed to manual techniques, significantly enhances fruit yield and reduces labor demands (Abrol et al., 2019; Wietzke et al., 2018; Gudowska et al., 2024; Ouvrard and Jacquemart, 2019). Despite high production volumes, the development of precision agriculture in strawberry farming in China is advancing slowly. Specifically, while bumblebees exhibit higher pollination efficiency, honeybees (*Apis mellifera*) are more commonly preferred by Chinese farmers due to their well-established husbandry practices and cost-effectiveness. Furthermore, in remote regions of China, traditional practices that favor honeybee pollination are deeply entrenched, accompanied by a noticeable lack of resources and knowledge for adopting bumblebee pollination. In this context, enhancing the understanding of honeybee natural pollination behaviors and devising strategies to optimize their efficiency are crucial for increasing strawberry yields in China (Gudowska et al., 2024).

Strawberry cultivars are propagated through cloning in nurseries to preserve the varietal traits, resulting in genetically identical plants within the same cultivar. While most strawberries are generally autogamous, relevant research suggested that many strawberry cultivars benefit from cross-pollination with pollen from other cultivars (Tuohimetsä et al., 2014). Additionally, the physiological characteristics of strawberry flowers also promote cross-pollination. Research has demonstrated that cross-pollination not only increases the average berry weight but also enhances the taste of strawberry fruit due to the Metaxenia mechanism (Dung et al., 2021). Therefore, cross-pollination is considered advantageous compared with self-pollination in commercial strawberry industry (Kakutani et al., 1993).

The primary objective of this study is to utilize the natural foraging behaviors of honeybees to enhance the pollination efficiency of strawberries, especially cross-pollination. According to our preliminary surveys in China, traditional strawberry interplanting has not considered the field design of greenhouses. For cultivation convenience, different varieties are typically planted in separate greenhouses, and farmers often lack a deep understanding of the behaviors of bees. Based on our preliminary research and related knowledge, we propose two hypotheses that could be used to improve pollination efficiency.

Firstly, we put forth the hypothesis that field design patterns exert a significant influence on cross-pollination performance and subsequent fruit weight in a greenhouse environment. In practical planting, the successful transfer of outcrossed pollen by honeybees in greenhouse strawberry cultivation is influenced by a complex interplay of variables, including the size and arrangement of the plant population, as well as the foraging behaviors of the honeybees (Barrett, 2003). However, there exists a notable lack in systematic research investigating the interactive effects of strawberry field design and honeybee movement on pollen transfer and fruit production (MacInnis and Forrest, 2020; Connelly et al., 2015; Opstad and Sønsteby, 2008). This is primarily due to the high cultivation costs involved and the challenges associated with tracking honeybee foraging and pollen grain trajectories (MacInnis and Forrest, 2020). Previous studies have only examined a limited range of planting patterns, with many aspects, particularly the pollination process, remaining unexplored (Madhavi et al., 2023). Given that honeybees serve as the primary pollinators in greenhouse strawberry cultivation in China, it is crucial to understand their unique flight patterns within strawberry fields (Skorupski et al., 2006). For instance, honeybees tend to consecutively forage on neighboring plants within bed. Gaining insights into how honeybee movement and interplanting patterns interact to impact crop pollination is essential for greenhouse strawberry cultivation (Baumann et al., 2002; Tsubo et al., 2005). To test our hypothesis, we proposed six representative field design patterns and conducted simulation experiments accordingly.

Secondly, we proposed an additional hypothesis that arranging strawberry cultivation into two separate batches within a greenhouse can stagger peak blooming periods, thereby reducing competition for bee pollination and enhancing fruit weight and yield. This hypothesis is grounded in the conclusions of our previous research. In botany, the physiological habits of flowers in different inflorescences exhibit variations in blooming periods (Malagodi-Braga and Kleinert, 2004). Strawberry inflorescences are arranged on a series of double branches, with a flower in the fork of each branch. In practical strawberry cultivation, it is customary to retain the primary and secondary inflorescences (Chagnon et al., 1989). These inflorescences have distinct blooming times, with the flowers in the primary inflorescence blossoming earlier, followed by the flowers in the secondary inflorescences, resulting in two distinct peak blooming periods during strawberry cultivation. Previous research (Cao et al., 2023) has demonstrated that high densities of blooming flowers during these peak periods can lead to intense competition for bee resources, subsequently diminishing pollination efficiency and fruit weight. Conversely, during the low blooming period, insufficient densities of blooming flowers may result in an inadequate pollen supply for bee transfer, further reducing pollination efficiency. In this study, we hypothesized the above cultivation strategy can effectively alleviate the negative impact caused by these problems, making it suitable for both single-cultivar and two-cultivar strawberry plantations in a greenhouse. We also intend to conduct simulation experiments to validate this hypothesis.

The simulation experiments conducted in this study were grounded in the utilization of previously strawberry pollination simulation model (SPSM) proposed by our team (Cao et al., 2023). To test the two hypotheses we proposed, the simulation model have been further optimized the model by incorporating the foraging behavior of honeybees and the characteristics of strawberry cultivars in this research. Specifically, we used the state machine method to model the flight behavior and state of honeybee within a greenhouse. The distinct advantage of simulation lies in its ability to avoid high cultivation costs while accurately tracking the trajectories of each pollen grain, flower, plant growth status, and bee activity (Bajcz et al., 2017). This capability provides reliable simulation results and serves as evidence for the effectiveness of various strawberry cultivation strategies. Furthermore, understanding the dynamics of bee pollination is a challenging task due to the multitude of factors involved, such as bee foraging behavior, planting patterns, and the spatial complexity of the greenhouse environment (Bethere et al., 2016). These factors interact over time and space, posing significant obstacles to comprehending bee pollination dynamics (Qu and Drummond, 2018). Quantitative understanding of these spatial interactions is difficult to achieve without the aid of simulation models (Qu et al., 2013), as bee behavior and plant floral distributions exhibit noticeable spatial heterogeneity and individual variation. In this paper, therefore, we modeled the foraging behaviors of honeybees within a greenhouse and employed the simulation method to investigate the influence of field design and staggered planting times on strawberry weight and yield. Our findings suggest that the most effective interplanting pattern for strawberries involves planting different cultivars in alternating rows within the same bed and staggering their planting time by approximately 5 days.

The contributions of this paper are as follows: 1) Improving the previously proposed SPSM model by incorporating multiple strawberry cultivars and simulating honeybee flight behavior; 2) Applying the state machine method and mathematical modeling to represent bee flight behavior in greenhouse environments; 3) Introducing an alternating planting strategy for different cultivars within the same bed to promote cross-pollination, thereby enhancing strawberry fruit yield; (4) Dividing strawberry plants into two batches and staggering their planting times to alleviate competition pressure for bee pollination.

## 2 Materials and methods

### 2.1 Strawberry simulation model

This study was based on the SPSM model previously proposed by our research team (Cao et al., 2023), which introduces a validated agent-based approach to simulating the bee-strawberry pollination process on the GAMA platform (Grignard et al., 2013). The simulation model is publicly available in GitHub: https://github.com/czh16/Greenhouse_Strawberry_Bee_Simulation.

**Figure 2:**
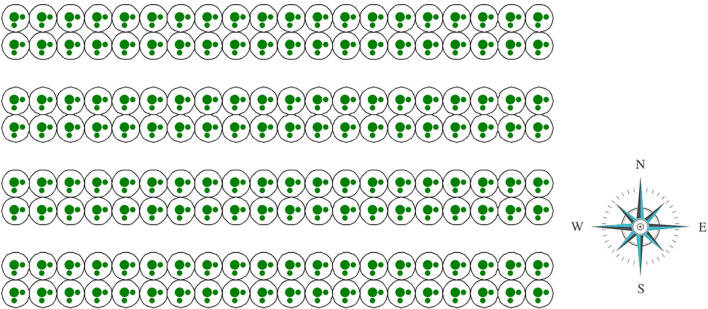
The visualization of simulation of greenhouse and strawberry plants.

The deployment and use of this model can reference previous research (Cao et al., 2023) and the tutorial in the GitHub repository; specific details will not be elaborated due to the length of the paper. This model accurately simulates the interactions between greenhouse strawberries and bees, producing results such as average berry weight, malformed fruit rate, and yield under various planting conditions. Notably, this model does not include analyses of taste, acidity, or other chemical properties, as these factors are difficult to quantify, their underlying mechanisms are unclear, and agent-based simulation is challenging. We acknowledge that the modeling process has overlooked certain subtle aspects of bee behavior, as these are challenging to capture through modeling methods. For example, the model assumes that each visit to a flower involves pollination process, whereas a very small portion of visits do not participate in pollination in reality (Barahona-Segovia et al., 2023). Comprehensive documentation, including source code and detailed annotations, is available in this repository. The visualization of simulation is illustrated in **Figure 2**.

The original model primarily focused on the cultivation of one single strawberry cultivar, specifically the Japanese *Beni hoppe* cultivar, chosen as a representative case for greenhouse cultivation because this cultivar is widely cultivated in China. However, in actual strawberry cultivation, numerous cultivars are grown, each with its own slight variations in habits and characteristics. We introduced an additional cultivar in simulation to emulate the interplanting process. The adjustable parameters of the model allow for fine-tuning to reflect the specific habits of different strawberry cultivars. To simplify the simulation, both cultivars share the same growth forms, floral morphologies, flowering densities, and blooming times. Moreover, we improved the model by incorporating the foraging behavior and state of honeybees and the simulation of cross-pollination processes between two strawberry cultivars within a greenhouse.

In general, all strawberry plants within the same cultivar are clones, meaning that each individual plant within the cultivar is genetically identical to others. Previous studies have revealed that many strawberry cultivars can benefit from cross-pollination, resulting in significantly improved berry quality compared to self-pollination (Tuohimetsä et al., 2014). This improvement can be attributed to the presence of self-incompatibility mechanisms in strawberries, which prevent fertilization by pollen from the same plant, thereby promoting genetic diversity within populations. Even when a pollen grain exhibits high viability and the stigma displays high receptivity, it does not necessarily guarantee successful pollen tube growth (Cao et al., 2023). In the simulation model, we employed the acceptance rate (acceptance rate = compatible pollen number/received pollen number) to represent the self-compatibility of a specific strawberry cultivar. By comparing the fruit weights under different conditions in relevant research and our previous study (Tuohimetsä et al., 2014), we set the acceptance rate to 90% for self-pollination and 95% for cross-pollination in simulation.

### 2.2 Honeybee foraging behaviours

Understanding the foraging habits of honeybees in a greenhouse is crucial for developing accurate and reliable simulation models (Chen et al., 2011). This understanding enables the enhancement of modeling accuracy and produces highly credible results. For instance, numerous studies have pointed out that weather conditions exert a significant influence on honeybee foraging behavior (Zhang et al., 2019). The most crucial weather condition is the temperature inside the greenhouse (Pathak et al., 2016). Additionally, due to the stable and favorable environment within the greenhouse, we ignore the influence of relative humidity on honeybee foraging behaviors. Honeybees tend to engage in foraging activities within a favorable temperature range of 15-25°C, while foraging activity declines when temperatures fall below 14°C or rise above 30°C (Abrol et al., 2019). On average, a bee visits approximately 2.5-3.8 flowers per minute, with an average interval of about 10 seconds between visits, and the bee typically spends around 10 seconds on a single flower (Chen et al., 2011).

In addition to these evident environmental factors and the inherent foraging habits of honeybees (Yang et al., 2015), the design of the field in a greenhouse can also influence the efficiency of bee pollination. This is primarily due to its effect on the flight paths followed by honeybees as they navigate between different strawberry cultivars. Strawberries are usually planted in raised bed, with two rows of strawberry plants per bed in a greenhouse.

Honeybees exhibit a centralized pattern of flower-visiting, guided by the principles of proximity, continuity, and repetition. Typically, honeybees can visit a succession of flowers on the same inflorescence. Once they have completed the visitation on one inflorescence, they will venture to nearby inflorescences to continue their exploration (Chen et al., 2011). Notably, studies have revealed that honeybees often display a strong sense of directionality (MacInnis and Forrest, 2020; Walters and Schultheis, 2009; Morris, 1993). When selecting their next destination, honeybees tend to move within the same bed of strawberry plants, with an approximately 0.85 probability of visiting flowers within that bed. It is rare for honeybees to traverse across different beds, as observed in an empirical study where out of 298 observed honeybees, only 14 ventured beyond their initial bed (Jilian et al., 2006). This behavior maximizes foraging efficiency and minimizes the chances of revisiting previously visited flowers which usually are of depletion (Collevatti et al., 2000). However, when neighboring rows contain open flowers, honeybees have a chance to visit these flowers in other rows.

**Figure 3:**
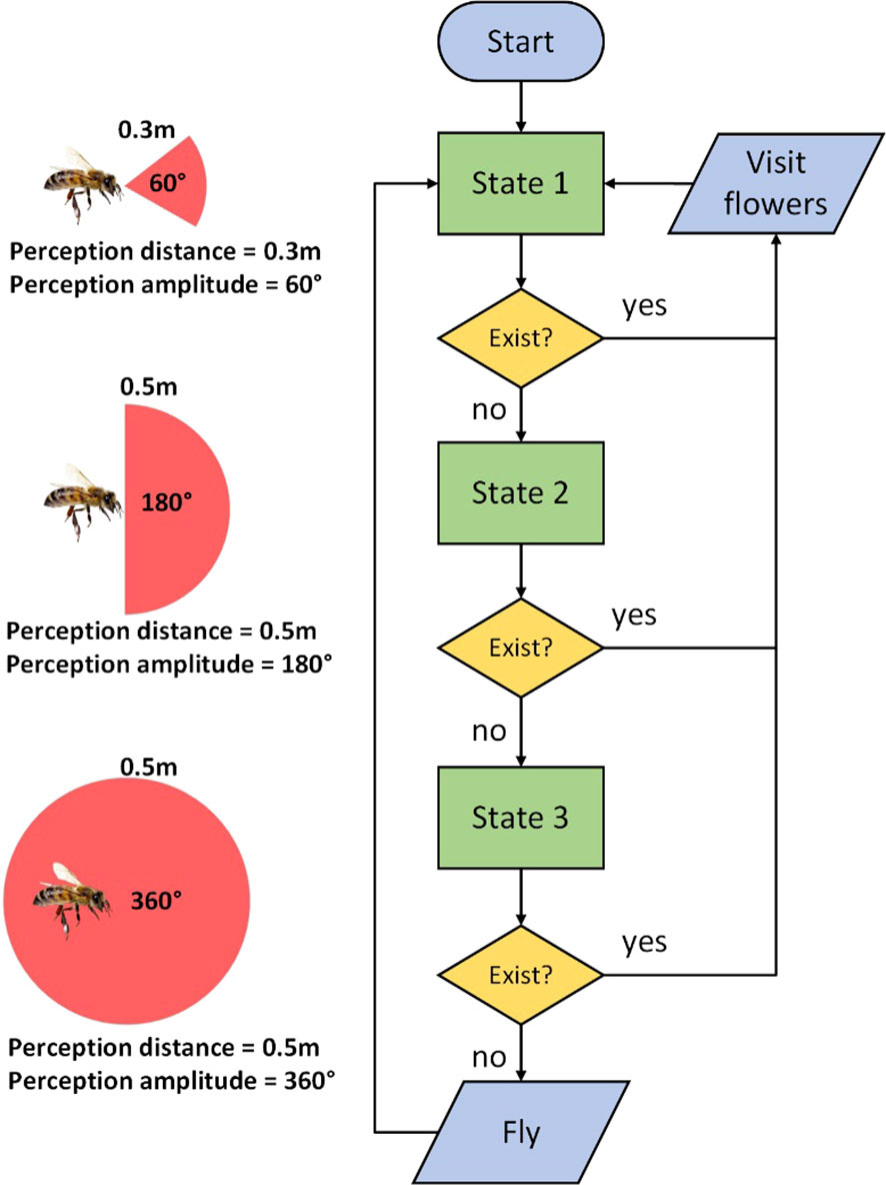
The transition chart between different states of of honeybees during foraging.

It is very important for the model to accurately express the flight patterns of honeybees. In response to the observed flight characteristics of honeybees in the strawberry greenhouse, we used the state machine method to model the bee flight behavior. In the simulation, each bee is represented as an Agent with three attributes while searching for flowers: direction, perception distance, and perception amplitude. Both perception distance and perception amplitude determine the bee’s perception range. Based on the flight behavior of honeybees, we categorized the honeybees during foraging into three states for simplification (Raine et al., 2006; Srinivasan, 2010), with perception distance and perception amplitude varying by state, while the direction is fixed along the line connecting two previously visited flowers (Srinivasan, 2011). The transitions between different states are illustrated in **Figure 3**.

State 1: The bee flies in the predetermined direction, with a perception distance of 0.3 m and a perception amplitude of 60°. If a blooming inflorescence is detected within the perception range, the bee randomly selects one and visit all flowers in this inflorescence, then immediately returning to State 1. If no blooming inflorescence is found, the bee enters State 2.

State 2: The perception distance is increased to 0.5 m and perception amplitude is widened to to 180° to expand perception range. If a blooming inflorescence is detected within the perception range, the bee randomly selects one and visit all flowers in this inflorescence, then immediately returning to State 1. If no blooming inflorescence is found, the bee enters State 3.

State 3: The perception amplitude is widened to 360° to further expand perception range. If a blooming inflorescence is detected within the perception range, the bee randomly selects one and visit all flowers in this inflorescence, then immediately returning to State 1. If no blooming inflorescence is found, the bee advances 1 m in a random direction to mimic the bee’s exploratory behaviors before returning to State 1.

This modelling method not only controls the directionality of the bee’s flight but also ensures a lower probability of the bee visiting neighboring rows. We set the parameters for perception distance and perception amplitude by continuously optimizing the simulation result through observing real bee flight paths and simulation outcomes. Additional information regarding the control of honeybees leaving the hive, returning to the hive, and their interaction with flowers can be found in the open-source codes provided in the **Supplementary Materials**, owing to constraints on manuscript length. The performance of this modeling approach is further validated by the results of subsequent Experiment 1.

### 2.3 The blooming time and fruit weight

The pollination process of strawberries by honeybees is highly intricate both in terms of spatial interactions between honeybees and flowers and temporal variations involving bee foraging and flower blooming. One important contributing factor is the differential blooming times and ovule quantities between primary and secondary inflorescences (Yoshida et al., 2012).

**Figure 4:**
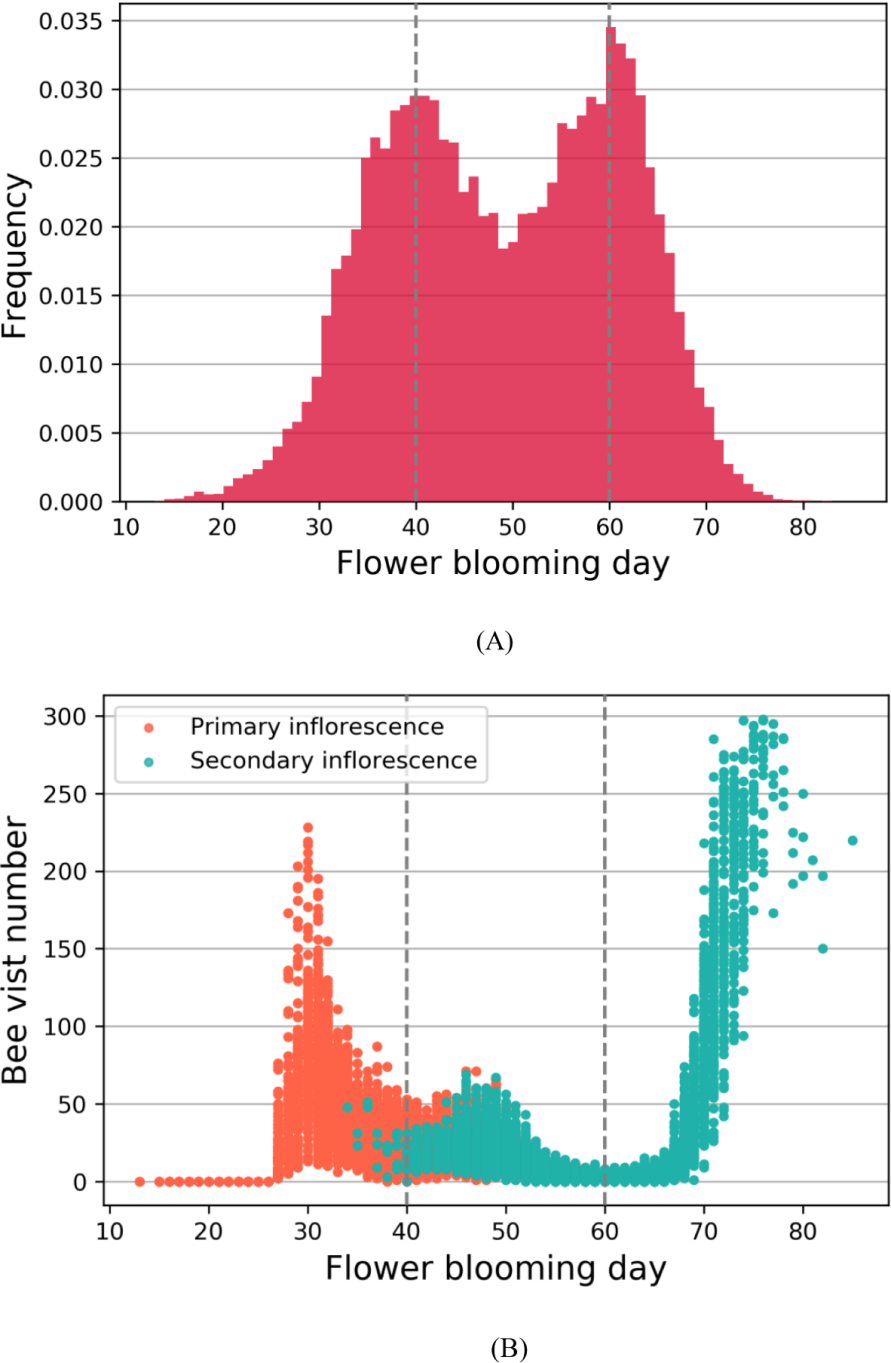
**(A)** The relationship between flower blooming day and frequency; **(B)** The relationship between flower blooming day and bee visit number. The dashed lines represent the two peak blooming periods at day 40 and day 60.

Previous simulation results have demonstrated that the blooming stage of flowers in the primary inflorescences tend to concentrate around day 40, while those in the secondary inflorescences exhibit a concentration around day 60, resulting in two distinct blooming peak periods although the overall blooming process of strawberries is continuous (Khammayom et al., 2022). During these peak periods, there is an obvious competition among flowers for the limited number of available honeybees, leading to reduced pollination efficiency and consequently lower fruit weight (Cao et al., 2023). Conversely, between the two peak periods, the low number of blooming flowers can result in insufficient pollen for transfer, thus also reducing pollination efficiency, as illustrated in **Figure 4**. This creates a situation where early-blooming (around day 30) flowers in the primary inflorescences and late-blooming (around day 70) flowers in the secondary inflorescences have more bee visits than needed. Moreover, flowers in primary inflorescences generally have a higher number of ovules compared to secondary inflorescences (Webb et al., 1974). This difference implies that primary flowers require a greater amount of pollen (Bethere et al., 2016). The complexity behind the pollination process makes it challenging to analyze using conventional statistical and machine learning methods.

## 3 Experiment design

In this study, we proposed three sets of experiments. The first set utilized simulation model to visualize and analyze honeybee flight trajectories. This analysis served to validate the performance of the proposed foraging behavior model, thereby establishing a foundational basis for subsequent investigations. The second set introduced six typical field designs and selected the optimal planting pattern in a simulated greenhouse based on results. The third set of experiments aimed to validate our hypothesis that dividing the strawberry plants into two batches and staggering their planting times can significantly enhance pollination efficiency.

These simulation experiments were conducted in a simulated greenhouse with standardized dimension of 80 m in length and 8 m in width, with raised beds of strawberry plants. The distance between two beds was 0.4 m, and each individual bed had a width of 0.6 m. Within each bed, two rows of strawberry plants were spaced approximately 0.24 m apart. The simulated greenhouse consisted of 24 rows (i.e., 12 beds), with 390 strawberry plants in each row. To ensure noticeable variations in experimental results, the simulation utilized a moderate bee density (10,000 honeybees foraging in the simulated greenhouse) because the effects of fruit yield improvement tend to diminish when honeybees are abundant, owing to saturation effects (Cao et al., 2023). For data analysis and hypothesis testing, we employed SPSS 26 software.

### 3.1 Strawberry simulation model

The first set of experiments aims at tracking the flight trajectory of honeybees and validating the performance of the proposed bee foraging model. The flight path of honeybees plays a crucial role in their foraging behavior and pollination efficiency (Svensson, 1990; Dos Santos et al., 2009; Grab et al., 2017; Zaitoun et al., 2006). Visualizing their flight trajectories can provide valuable insights into understanding bee movement patterns and model validation. In this set of experiments, we used the improved simulation model to analyze the flight trajectories of honeybees and compared them with those of real honeybees in a greenhouse. By incorporating various factors such as greenhouse environmental conditions, planting field patterns, and bee behaviors, we can reconstruct honeybee flight paths in a virtual environment. Furthermore, these results allow us to visually identify specific regions of interest where honeybees frequently visit during flower selection (Qu et al., 2013).

The advantage of simulation lies in the ability to accurately record the flight trajectories of each individual bee and utilize the statistical results from all honeybees to present the preferred zone for the next flower. To focus our study on bee flight patterns and exclude the influence of cross-pollination, we selected a single strawberry cultivar for this experiment. Specifically, we chose the 40th day of simulation, a period during which a larger number of flowers are in bloom and honeybees are highly active in foraging. To validate the results, we employed two methods: 1) by observing the flight trajectories of individual honeybees and comparing them with those of real honeybees in a greenhouse; 2) by analyzing the flight trajectories of all honeybees to statistically determine the honeybees’ preferred zone for selecting the next flower, and comparing these findings with relevant literature.

The visualization and analysis of honeybee flight paths can provide valuable insights into their interaction with strawberry plants and contribute to understanding of pollination dynamics, which can serve as the foundation for the next experiments.

### 3.2 Honeybee foraging behaviours

The second set of experiment was designed to evaluate the optimal field design within a simulated greenhouse. Six typical field design patterns were proposed, each with the same specifications and dimension as described earlier. The field design pattern within the greenhouse has an impact on the pollination process of honeybees between different strawberry cultivars, thereby affecting the yield of the strawberry fruits. By comparing the average fruit weight and malformed fruit rate (fruits with ovule fertilization rate below 87%) in the experimental results (Ariza et al., 2012), we can analyze which field design is more conducive to promoting bee cross-pollination. In the simulation, two strawberry cultivars, A and B, were planted in the greenhouse. For simplicity, we assumed that both varieties A and B have similar growth forms, floral morphologies, flowering densities, and bloom times. This means that the two varieties have similar peak and valley flowering times.

**Figure 5:**
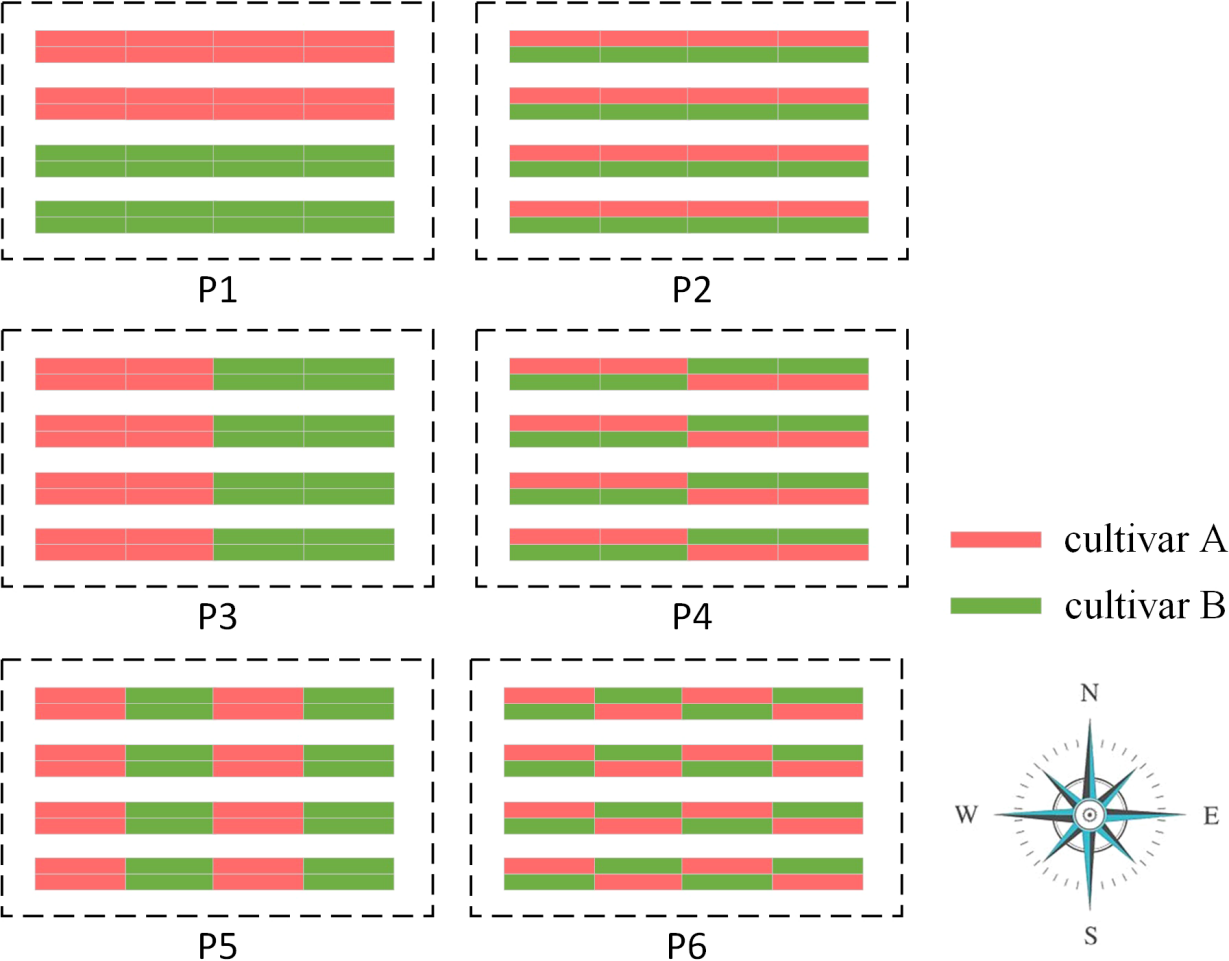
Six typical field design patterns. Red for cultivar A and green for cultivar B.

By consulting experts in Shandong Agricultural University on actual strawberry cultivation experience and relevant research (MacInnis and Forrest, 2020), we designed six typical planting patterns as representatives, labeled as P1, P2, P3, P4, P5, and P6, as shown in **Figure 5**. Each planting pattern scenario was simulated 10 times (i.e., N = 10). In P1, the greenhouse was divided into two blocks in the vertical direction (i.e., north-south direction), with cultivar A planted in the upper block and cultivar B planted in the lower block; In P2, the different cultivars are planted in alternating rows within the same bed; In P3, the greenhouse was divided into two blocks in the horizontal direction (i.e., east-west direction), with cultivar A planted in the left block and cultivar B planted in the right block; In P4, the greenhouse was divided into two horizontal blocks, and the planting pattern in each block is the same as in P2, but with the strawberry cultivars reversed; In P5, the greenhouse was divided into four horizontal blocks, with the same cultivar planted in each block and different cultivars planted in adjacent blocks; In P6, the greenhouse was divided into four horizontal blocks, with different cultivars planted in different rows within the same bed. These six planting patterns were chosen to represent different arrangements and combinations of strawberry cultivars within the greenhouse.

By simulating these patterns and comparing the results, we can determine which field design most effectively promotes cross-pollination and enhances fruit quality. We evaluate fruit quality based on four indicators: average berry weight, malformed fruit rate, ratio of foreign pollen, and yield.

We proposed an additional set of experiments to analyze whether there were significant differences in average fruit weight among different positions within each planting pattern. We considered the greenhouse east-west direction as the X-axis and the north-south direction as the Y-axis. The X-axis contained 390 columns of straw-berry plants (i.e., 390 groups), while the Y-axis contained 24 rows of strawberries (i.e., 24 groups). We randomly chose 24 rows of strawberries in the greenhouse as representatives for the analysis. This analysis of significant differences helps us ascertain whether the distribution of fruit weight is uniform along both the X-axis and Y-axis, thereby enhancing our understanding of the field design.

### 3.3 The blooming time and fruit weight

The third set of experiments was to test the second hypothesis that dividing the strawberry plants into two batches (i.e., batch A and batch B) and staggering their planting times can effectively utilize bee resources in both single-cultivar and two-cultivar strawberry plantations. This strategy aims to achieve a more uniform distribution of flower blooming days, which can help reducing competition for pollination service during the peak blooming period and address the issue of insufficient pollen during the valley blooming period.

We chose the P3 pattern to conduct experiments because it is simple to implement in an actual greenhouse and, based on the results of the second set of experiment, the cross-pollination efficiency of P3 pattern was found to be low with significant improvement of pollination efficiency, which is beneficial for observing and comparing the experiment results. In two-cultivar plantations, we divided the strawberry seedlings into two batches based on the cultivars. In single-cultivar plantations, the straw-berry seedlings were randomly divided into two batches.

Batch A was planted first, then batch B was planted with a N days lag. The quality of strawberry fruits in this scenario was observed and analyzed. In actual strawberry greenhouse planting, a slight delay in the planting schedule is acceptable. Based on practical planting experience, we set N to 0 and 5 in the simulation as setting N too large may interfere with the actual cultivation of strawberries. Furthermore, to comprehensively analyze the impact of this staggering planting time strategy, we proposed experiments to analyze the frequency distribution of flower blooming dates for both batches and the entire population. We also recorded and compared the peak and valley values of the distributions, along with their respective occurrence days. This allowed us to gain deeper insights into the effects of delayed planting on flower blooming patterns.

## 4 Experiment results

### 4.1 Experiment I

**Figure 6:**
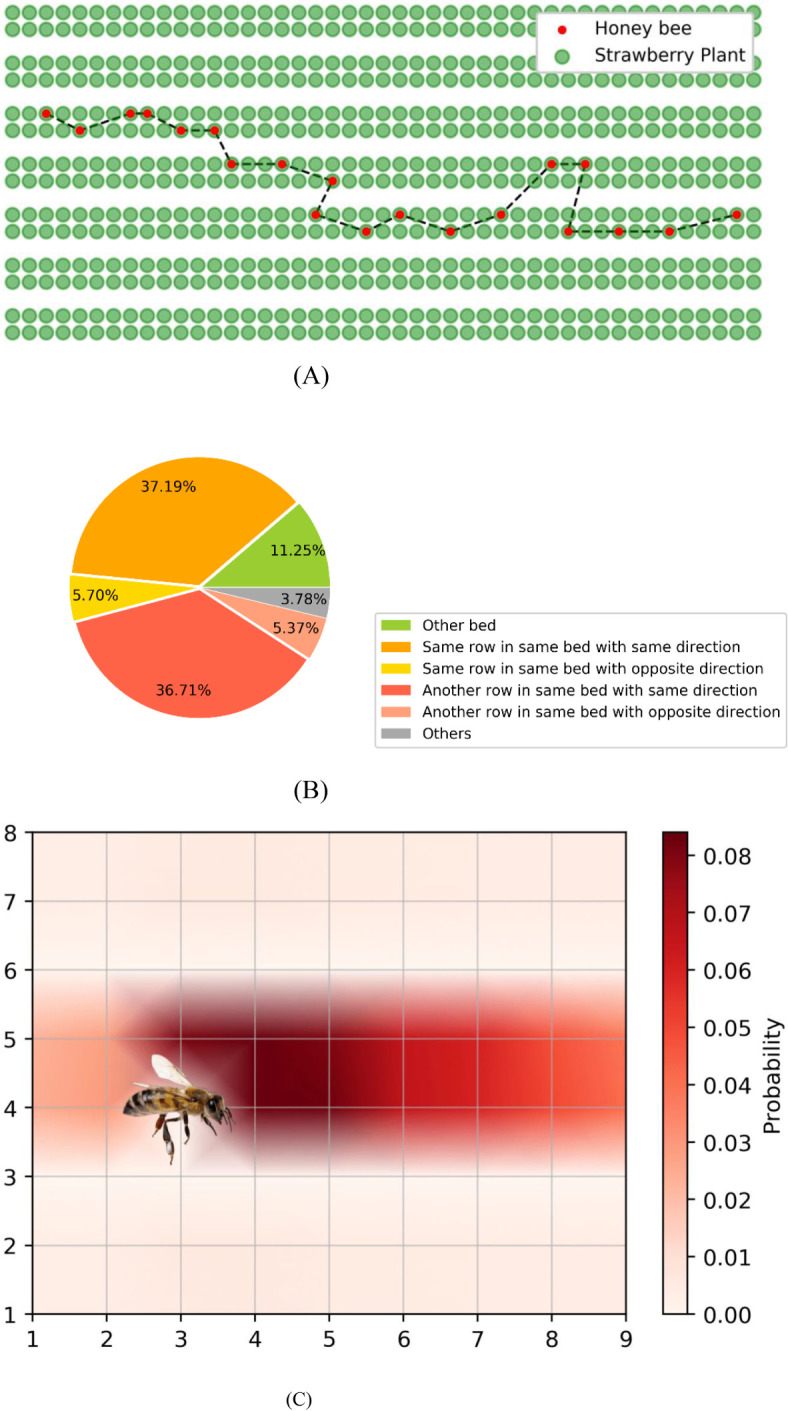
**(A)** A typical honeybee flight trajectory in the simulation. **(B)** Likelihood of the next flower location that honeybees visit, categorized by different regions. **(C)** Density plot of next flower location that honeybees visit. Each grid intersection represents a strawberry plant and the direction of bee’s flight is to the right.

We tracked the flight trajectory of all honeybees in the greenhouse on the 40th day and collected data on their next flower selection after completing the current flower visit in the simulation. The flight trajectory of an individual bee selected is depicted in **Figure 6A**. It is evident that the simulated bee tends to move between strawberry plants within the same bed. Moreover, when open flowers are present in adjacent beds, honeybees can enter other beds to visit the blooming flowers. This simulation result aligns with observations from actual greenhouse experiences and has been validated by planting experts.

Then we counted the flight trajectories of all honeybees on the 40th day of simulation. **Figures 6B, C**, present the likelihood of simulated honeybees’ next flower location after completing current pollination task. We categorized the flight trajectories into six types based on position of flower to visit and current flight directions, with “others” in **Figure 6B** representing honeybees returning to the hive after foraging. It can be observed from the simulation results that when flying among beds in a greenhouse, there is an 11.25% probability of crossing to other beds, a 42.89% probability of visiting flowers in the same row of the same bed, and a 42.08% probability of visiting flowers in another row of the same bed. The results indicate that honeybees flight trajectories in the simulation exhibit strong directionality. After consulting with experts, reviewing relevant literature, and conducting observations in an actual greenhouse using industrial cameras, we find the statistical results of honeybee flight trajectories align with empirical data and the patterns summarized in relevant literature, which further validates the reliability of the model. We acknowledge that, due to the high stochasticity of bee flight and the current challenges in quantifying it using suitable metrics, this evaluation method represents a practical compromise.

### 4.2 Experiment II

The results of the six field planting patterns in Experiment II are presented in **Table 1**. We repeated this experiment 10 times (N = 10). The data indicated significant differences in fruit quality among the different field planting patterns (p< 0.05) based on ANOVA analysis.

**Table 1:** Impact of planting patterns on strawberry fruit quality based on ANOVA test.

Planting patterns	Average berry weight (g)	Average malformed fruit rate (%)	Average ratio of foreign pollen (%)	Average yield (kg/ $m^{2}$ )
P1	15.30 ± 0.11 b	19.89 ± 0.80 b	16.25 ± 0.67 b	3.31 ± 0.06 b
P2	15.64 ± 0.17 a	11.17 ± 0.92 c	49.38 ± 0.82 a	3.38 ± 0.06 a
P3	15.14 ± 0.16 c	23.19 ± 0.72 a	2.02 ± 0.72 d	3.25 ± 0.05 c
P4	15.64 ± 0.15 a	11.20 ± 0.94 c	49.36 ± 0.74 a	3.38 ± 0.06 a
P5	15.17 ± 0.14 c	23.35 ± 0.50 a	5.82 ± 0.52 c	3.26 ± 0.05 c
P6	15.66 ± 0.12 a	10.96 ± 0.66 c	49.40 ± 0.60 a	3.38 ± 0.05 a

Means ± SE within a column followed by different letters were significantly different (P > 0.05, with N = 10 replicates for a single set of simulation experiments).

Regarding the average berry weight, the results demonstrate that P2, P4, and P6 yield high outcomes with no significant differences observed between them. The average berry weights for these patterns were 15.64 ± 0.17 g, 15.64 ± 0.15 g, and 15.66 ± 0.12 g, respectively. In contrast, the simple planting pattern P1 exhibited a lower average fruit weight of 15.30 ± 0.11 g. Among the six patterns examined, P3 and P5 showed the lowest fruit weights of 15.14 ± 0.16 g and 15.17 ± 0.14 g, respectively.

Similar conclusions can be drawn based on the average rate of malformed fruit and average yield. Among the six patterns, P2, P4, and P6 demonstrated optimal outcomes, with no significant differences observed between them. The average rates of malformed fruit for these patterns are 11.17 ± 0.92%, 11.20 ± 0.94%, and 10.96 ± 0.66%, respectively. The average yields for these patterns are 3.38 ± 0.06, 3.38 ± 0.06, and 3.38 ± 0.05 kg/m^2^, respectively. In contrast, P3 and P5 exhibited the highest rates of malformed fruit and lowest average yield, indicating them as the two worst planting patterns. Moreover, P1 achieved moderate results with an average rate of malformed fruit at 19.89 ± 0.80% and an average yield of 3.31 ± 0.06 kg/m^2^.

To accurately assess the cross-pollination efficiency of each planting pattern, we conducted an analysis of the average ratio of foreign pollen for flowers under each pattern. A higher value indicated a greater efficiency of cross-pollination facilitated by honeybees. Furthermore, this value may serve as an indirect indicator of improved flavor quality. Referring to **Table 1**, it was evident that pattern P2, P4 and P6 demonstrated the highest cross-pollination efficiency with no significant differences observed, and each flower receiving approximately half of pollen grains from the other strawberry cultivar (49.38 ± 0.82%, 49.36 ± 0.74% and 49.40 ± 0.60% respectively). Following them, P1 experienced a significant decline in the ratio of foreign pollen compared to the preceding three patterns (16.21 ± 1.05%). The two worst planting patterns, P3 and P5, exhibited extremely low cross-pollination efficiency, with values of 2.02 ± 0.72% and 5.82 ± 0.52% respectively. However, there was a significant difference between the results, with P3 showing the lowest cross-pollination efficiency. This finding suggests that the average ratio of foreign pollen is a more precise evaluation metric compared to average berry weight and average malformed fruit rate.

In conclusion, although the experimental results of P2, P4, and P6 demonstrated similarities, it is apparent that P2 stands out with its simple design and suitability for practical greenhouse cultivation. Consequently, based on the assessment using the average ratio of foreign pollen, we conclude that P2 represents the optimal planting pattern, while P3 is considered the least favorable option.

The experimental results of the significance test between the average fruit weight of strawberry plants and their positions in the greenhouse are presented in **Table 2**. Under the P1 scenario, no significant difference in fruit weight distribution was observed along the X-axis, while a significant difference was evident along the Y-axis, as illustrated in **Supplementary Figure S1A**. The fruit weight increased as the strawberry plants were positioned closer to the center. In the P2, P4, P5, and P6 scenarios, there were no significant differences in fruit weight distribution along both the X-axis and Y-axis. However, in the P3 scenario, a significant difference was observed along the X-axis, as shown in **Supplementary Figure S1B**, while no significant difference was found along the Y-axis. Notably, despite the similarity between P3 and P5, it was discovered that the fruit weight distribution along the X-axis was not significantly different in the P5 scenario, distinguishing it from P3, as depicted in **Supplementary Figure S1C**.

**Table 2:** The results of the significance test in X-axis and Y-axis.

Planting patterns	X-axis	Y-axis
P1	/	√
P2	/	/
P3	√	/
P4	/	/
P5	/	/
P6	/	/

The symbol √ in the table indicates a significant difference between the groups of strawberry plants in that direction, while the symbol / indicates no significant difference in that direction.

### 4.3 Experiment III

Firstly, we conducted experiments to explore the two-cultivar plantation scenario. We repeated this experiment 10 times (N = 10). We selected planting pattern P3, as mentioned earlier, as the representative. The strawberry seedlings were divided into two batches based on the cultivars, and their planting times were staggered by N days which was set to 0 and 5.

The experimental results based on T-test presented in the upper half of **Table 3** demonstrate significant differences in fruit quality under different N values (p< 0.05). For the average berry weight, when N was set to 0, the average fruit weight was 15.14 ± 0.18 g, whereas when N was set to 5, the average fruit weight increased to 15.39 ± 0.15 g. The observed significant difference indicated that the delayed planting strategy can enhance the individual fruit weight of strawberries. Regarding the average malformed fruit rate, when N was set to 0, the average rate of malformed fruit was 23.19 ± 0.72%, whereas when N was set to 5, the average rate decreased to 18.25 ± 0.60%. The average yields are 3.25 ± 0.05 and 3.31 ± 0.06 kg/m2, respectively. The numerical comparison illustrated that the delayed planting strategy can increase yield by approximately 2%.

**Table 3:** Impact of the N-days delay in two plantation patterns based on T-test.

N	Average berry weight (g)	Average malformed fruit rate (%)	Average yield (kg/ $m^{2}$ )	Plantation pattern
0	15.14 ± 0.18 b	23.19 ± 0.72 a	3.25 ± 0.05 b	two-cultivar
5	15.39 ± 0.15 a	18.25 ± 0.60 b	3.31 ± 0.06 a	two-cultivar
0	15.12 ± 0.17 b	24.75 ± 0.60 a	3.24 ± 0.05 b	single-cultivar
5	15.33 ± 0.15 a	19.31 ± 0.57 b	3.30 ± 0.05 a	single-cultivar

Means ± SE within a column followed by different letters were significantly different (P > 0.05, with N = 10 replicates for a single set of simulation experiments).

Secondly, we tested the scenario in which only one strawberry cultivar was planted in a greenhouse. In this scenario, we also set the day delay N for single-cultivar plantation to 0 and 5. The experiment results are shown in the lower half of **Table 3**.

The data presented indicate that the results from single-cultivar plantations were consistent with those observed in two-cultivar plantations. Significant differences in average berry weight, rate of malformed fruit, and yield were noted when N was set to 0 and 5, respectively. Specifically, when N was set to 0, the average berry weight was 15.12 ± 0.43 g, with a malformed fruit rate of 24.75 ± 0.60% and a yield of 3.24 ± 0.05 kg/m². In contrast, with N set to 5, the average berry weight slightly increased to 15.27 ± 0.32 g, the yield rose to 3.30 ± 0.05 kg/m², and the malformed fruit rate decreased to 19.31 ± 0.57%. These results provide strong evidence for the applicability and effectiveness of the proposed staggering planting time strategy in both single-cultivar and two-cultivar plantations.

**Figure 7:**
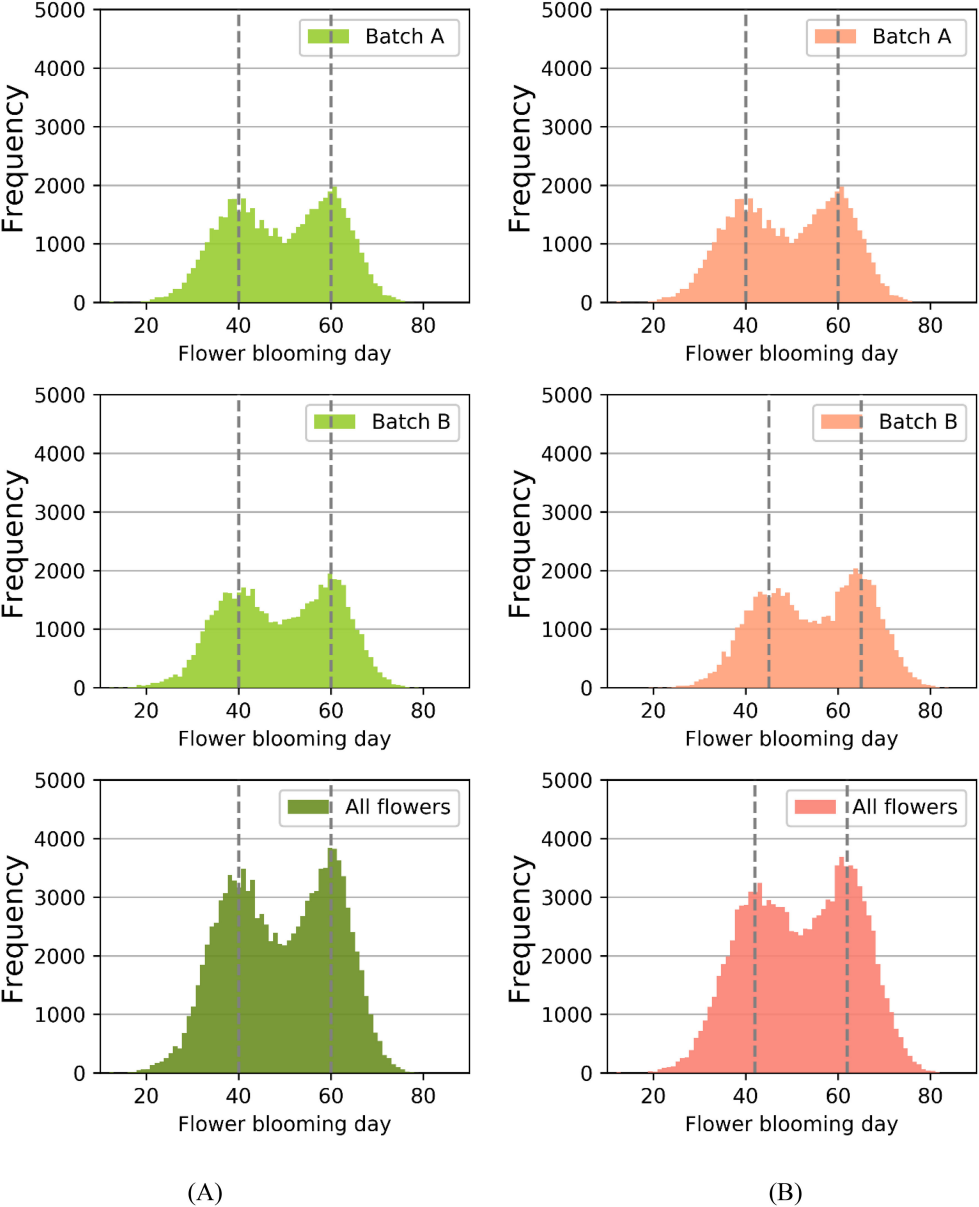
**(A)** When N was set to 0, the frequency distributions of flower blooming dates for batch A, batch B, and all strawberry flowers. **(B)** When N was set to 5, the frequency distributions. The dashed lines represent the peak blooming periods.

**Figure 7** depicted the frequency distributions of flower blooming dates for batch A, batch B, and the entire strawberries under two different scenarios: N set to 0 and N set to 5. In the simulation, the total number of strawberry flowers considered was 112,320, which was calculated as the product of 390 columns, 24 rows, and 12 flowers per plant. **Figure 7A** illustrated the scenario with N set to 0, while **Figure 7B** represented the scenario with N set to 5.

We first analyzed the blooming data distribution for each individual batch. Regarding batch A, the setting of N had no impact on the blooming date distribution. However, for batch B, when N was set to 5, the flowering dates were on average delayed by approximately 5 days compared to the scenario with N set to 0.

In the subsequent analysis, we investigated the blooming data distribution of all strawberry flowers, as illustrated in **Table 4** and the two small graphs in the third row of **Figure 7**. For N set to 0, the first blooming peak occurred on day 40 with a frequency of 3,489, followed by a valley on day 49 with a frequency of 2,205, and then a second peak on day 60 with a frequency of 3,825. Conversely, with N set to 5, the first blooming peak appeared on day 43 with a frequency of 3243, the valley emerged on day 52 with a frequency of 3,243, and the second peak manifested on day 63 with a frequency of 3,540. Comparing these two scenarios, we observed that the implementation of the planting strategy resulted in a delay of approximately 3 days in both the peak and valley frequencies of blooming dates for all flowers. Additionally, it was observed that the values for both blooming peaks were lower, while the valley value was higher compared to the scenario with N set to 0. These findings provided compelling evidence that the staggering planting time strategy successfully balanced the frequency distributions of blooming peaks and valleys, achieving a more balanced and even distribution of blooming dates.

**Table 4:** The days of peak and valley in the blooming distribution.

*N*	First peak	valley	Second peak
0	3489 (40^th^)	2205 (49^th^)	3825 (60^th^)
5	3243 (43^th^)	2349 (52^nd^)	3540 (63^th^)

## 5 Discussion

In this paper, we enhanced the previously proposed SPSM model by integrating multiple strawberry cultivars and simulating honeybee flight behavior. Our findings indicate that implementing an alternating planting strategy for different cultivars within the same bed, along with dividing strawberry plants into two batches and staggering their planting times, can effectively boost strawberry yield.

The focus of this simulation study was to fully utilize the foraging behavior of honeybees to enhance the pollination efficiency for greenhouse strawberries. The proposed optimal field design and staggering planting time are two cultivation strategies that can improve strawberry yields in greenhouse strawberry production in China. While these cultivation practices may have been adopted by some farmers in the field, a theoretical foundation is lacking. This study employs simulation techniques to theoretically validate their feasibility.

The results of Experiment I revealed that simulated honeybees tend to exhibit directional flight patterns within a single bed in the strawberry greenhouse, and they showed an almost equal probability of visiting flowers in two rows of the same bed. The statistical results of honeybee flight trajectories align with empirical data and the patterns in relevant literature, which validates the the proposed model. In practical planting, this directional behavior is beneficial as it prevents honeybees from revisiting previously pollinated flowers, reduces flight randomness, and ultimately enhances pollination efficiency. Additionally, when open flowers are present in adjacent beds or behind the honeybees, and these flowers are within honeybees’ perception range, there is a possibility for the honeybees to visit them. Understanding and utilizing these flight characteristics of honeybees can lead to improvements in greenhouse strawberry planting strategies, which is the foundation for the following investigations in this study.

The results of Experiment II demonstrated the superiority of the P2 interplanting pattern when two strawberry cultivars were simultaneously grown in the greenhouse. P2 stood out as the most optimal design due to its simplicity and the effective promotion of cross-pollination, capitalizing on the honeybee natural tendency to consecutively forage on neighboring plants within a bed. The key feature of P2 lies in planting two different strawberry cultivars in separate rows within the same bed. This strategic arrangement maximizes pollination efficiency.

Although P4 and P6 achieved similar fruit quality and yield as P2, they adopted a more complex field design initially intended to increase interplanting between different cultivars and thereby enhance cross-pollination efficiency. However, simulation results showed that this intricate design did not significantly improve cross-pollination efficiency, mainly because honeybees exhibited an almost equal probability of visiting flowers in two rows of the same bed (Svensson, 1990; Dos Santos et al., 2009). The complex designs of P4 and P6 would increase labor costs during seedling planting and raise management costs in cultivation management. Consequently, we regarded P4 and P6 as less practical planting patterns compared to P2.

The experimental results of P1 and P3 are unexpected. Initially, we expected that P3 would outperform P1 since honeybees tend to fly along the same bed. However, the results indicated that P1 exhibited better performance. Upon tracking the flight trajectories of honeybees and pollen flow, we discovered that this discrepancy was due to the bed length being much larger than its width in a greenhouse. In P3 scenario, when honeybees fly along the same bed, pollen extraction and deposition predominantly occurred within a single cultivar block, leading to reduced cross-pollination efficiency. Conversely, in P1 scenario, despite the honeybee’s inclination to fly along the same bed, there remained a chance to transition to the adjacent block and access another strawberry cultivar, thereby facilitating cross-pollination.

Our findings provide a plausible explanation for an intriguing phenomenon observed in previous research. In the study conducted by Wiebke et al. (Kämper et al., 2022), they utilized the P1 planting pattern for strawberry cultivation in the field, with significantly larger blocks compared to those in this paper. Surprisingly, they found a low probability of cross pollination between different cultivars but failed to offer a reasonable explanation for this uncommon observation. Based on the results of our study, we attribute this phenomenon to honeybees’ preference for flying within the same bed, resulting in minimal chances of traversing into other blocks to access different cultivars. Nonetheless, this does not negate the significant role of honeybees in cross-pollination in strawberries.

P3 and P5 were considered relatively less favorable planting patterns in the simulation. Although there were no statistically significant differences for average berry weight and average malformed fruit rate, analyzing the average ratio of foreign pollen and the fruit weight distribution along the X-axis leads to the conclusion that P5 slightly outperforms P3. Additionally, the assessment of the average ratio of foreign pollen is challenging to conduct in actual cultivation due to the high costs involved, highlighting an important advantage of SPSM model on which various simulation experiments can be conducted. We believe that the reason for P5’s superiority over P3 lies in its more intricate design, featuring four alternating blocks for planting. Consequently, compared to P3, honeybees find it easier to traverse between different blocks in P5, thereby promoting cross-pollination.

This simulation model allows for precise tracking of every pollen movement within the greenhouse, facilitating a detailed analysis of the average ratio of foreign pollen and offering valuable insights into field design (Cao et al., 2023). The experimental findings demonstrated that the distribution of strawberry fruit weight along the Y-axis in P1 and the X-axis in P3 is not uniform, indicating varying degrees of cross-pollination. In both P1 and P3 scenarios, the strawberry fruits located at the boundaries between two blocks exhibit relatively higher weight, while those farther from the boundaries display relatively poorer weight. Consequently, ensuring adequate interaction between the two strawberry cultivars in the field design becomes a crucial factor in enhancing pollination efficiency, thereby contributing to the superior performance of P2, P4, and P6.

The results of Experiment III demonstrated the effectiveness of the proposed staggering planting time strategy in both two-cultivar and single-cultivar plantations. The experimental outcomes indicated that in both scenarios, delaying the planting of batch B strawberries by 5 days can lead to a more efficient utilization of bee resources, resulting in a significant increase in the average berry weight and a reduction in the average rate of malformed fruits (by 4.94% and 5.44% respectively). It is important to dynamically adjust the value of N for the delay based on actual planting conditions during practical cultivation, while ensuring it does not impede regular planting activities (Nielsen et al., 2002; Hussain et al., 2012).

The experimental results validated our proposed hypothesis that the different habits of two inflorescences in strawberries lead to two blooming peaks and a valley during the continuous blooming process. This uneven distribution results in the inefficient utilization of bee resources. During peak period, there are numerous open flowers, leading to high competition for bee resources, and the greenhouse provides an abundant amount of pollen for pollination. In contrast, during the valley period, there are fewer open flowers, resulting in reduced competition for bee resources, but the greenhouse offers limited pollen for pollination. Hence, these three factors exhibit a complex relationship that is challenging to analyze using ordinary statistical or machine learning methods. However, simulation models can effectively analyze the output of such complex relationship networks.

In the paper, we tracked the blooming status of 112,320 flowers inside the greenhouse in detail based on the SPSM model. The experimental results confirmed that the staggering planting time strategy, which involves staggering the blooming peaks of batch A and batch B by five days, can balance the distribution of blooming peaks and valleys among all flowers (Cao et al., 2023). This approach reduces the number of blooming flowers during peak stages and increases the number of blooming flowers during the valley stages, ultimately enhancing the efficiency of bee resource utilization. We believe that the effectiveness of this strategy lies in the fact that the daily supply of bee resources inside the greenhouse remains constant. By implementing the staggering planting time strategy, the overall blooming period of strawberry flowers in the greenhouse is extended by approximately 3 days, enabling the utilization of more bee resources for pollination activities. This approach represents a typical strategy of effectively exchanging time for resources.

The study presented in this paper still has some limitations. When modeling the complex foraging behavior of honeybees, we simplify the process by categorizing them into three states and omitting some behavioral details for simplicity. However, simulation results indicate that this simplified modeling method can basically describe the flight characteristics of honeybees. Additionally, to simplify the simulation process, we assumed the same growth characteristics for both strawberry cultivars. Such simplification is common in computer simulations and aids in the model construction. Moreover, to achieve more noticeable improvement effects, we chose the scenario with a slightly lower number of honeybees for simulation. As a result, the actual enhancement in fruit yield based on the proposed planting strategies may be lower than what was observed in the simulation. Our future research will focus on incorporating more physiological parameters of various strawberry cultivars into the SPSM model. These parameters may include the growing degree days (GDD), the number of ovules, the number of inflorescences, pollen viability and so on (Lata et al., 2018; Antunes et al., 2010), thereby providing a richer dataset for the strawberry cultivation community.

## 6 Conclusions

This research investigated the impact of strategic interplanting and bee-pollination patterns on enhancing strawberry yield within greenhouse settings, employing simulation methodologies to study optimal cultivation techniques. The core objective was to use the natural foraging behaviors of honeybees to improve the cross-pollination process, thereby augmenting fruit quality and yield.

Our findings indicate that adopting an alternating planting approach for different cultivars within the same bed effectively facilitates cross-pollination, resulting in improvements in strawberry fruit weight. Additionally, dividing the strawberry plants into two batches and staggering their planting time approximately 5 days helps mitigate the pressure of competition for bee pollination during peak blooming period, consequently contributing to enhanced fruit weight and yield. These findings offer valuable cultivation suggestions for farmers in some remote areas in China who still rely on honeybees as primary pollinators.

## Data Availability

The datasets presented in this study can be found in online repositories. The names of the repository/repositories and accession number(s) can be found in the article/**Supplementary Material**.
