# Supplementary material for: Strategies to enhance greenhouse strawberry yield through honeybee pollination behavior: a simulation study
Source: Front Plant Sci. 2024 Dec 5;15:1514372. doi: 10.3389/fpls.2024.1514372 (PMC11655200; doi:10.3389/fpls.2024.1514372)
Supplement: Supplementary file 1 [file DataSheet1.docx]

Supplementary Material


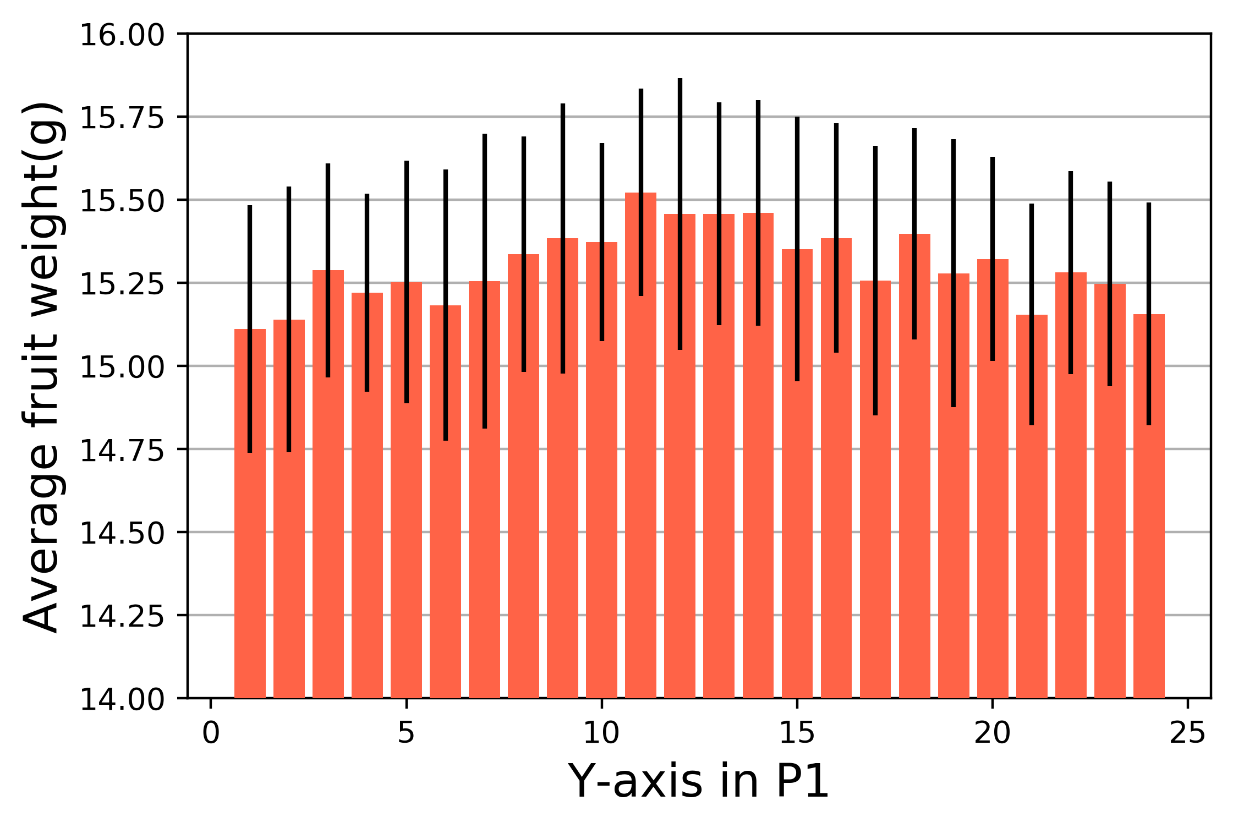


(A)


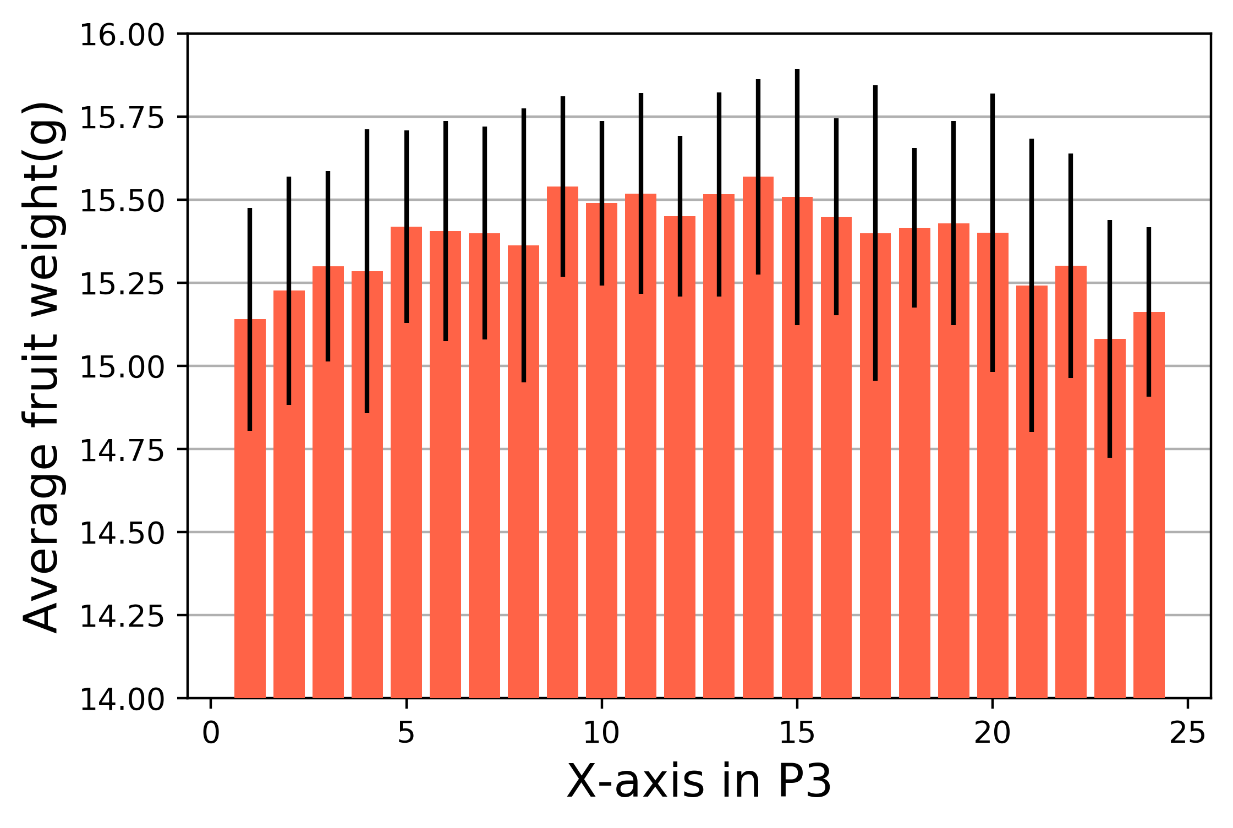


(B)


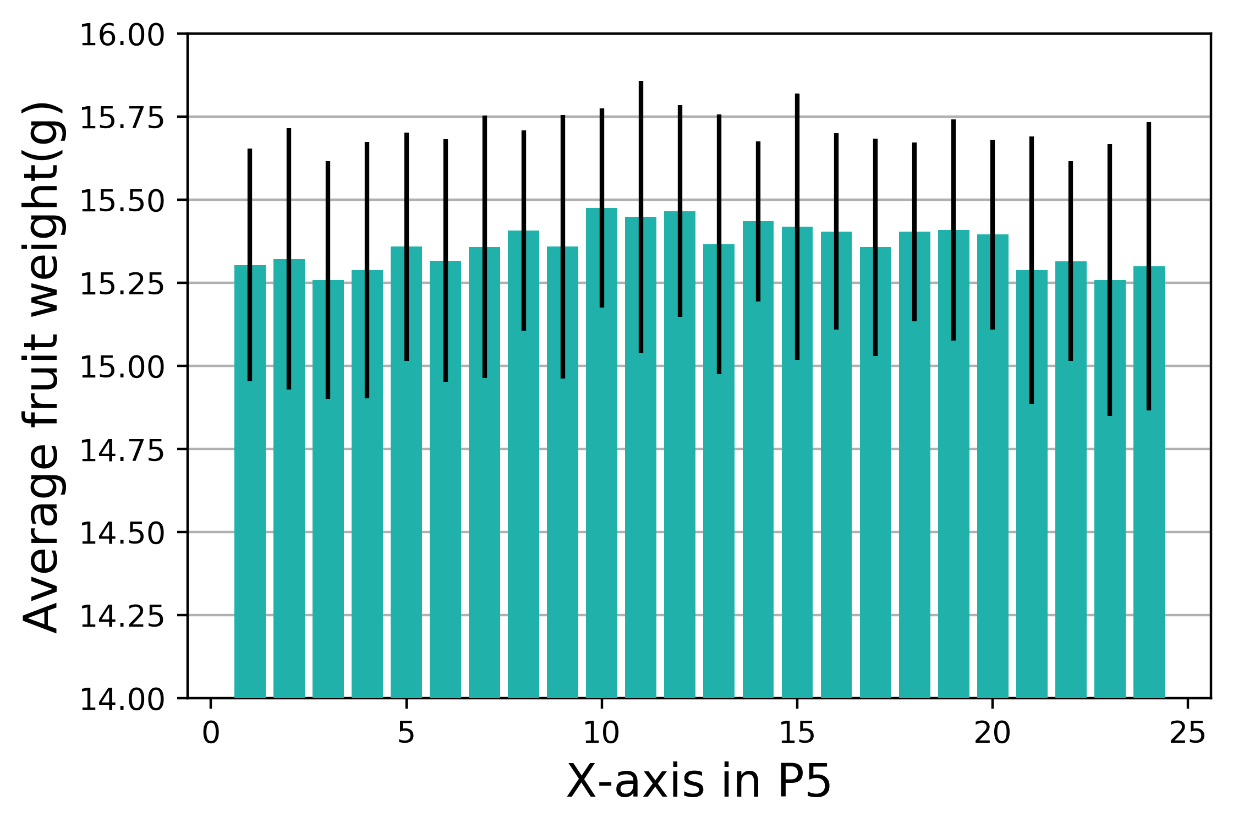


(C)

**Supplementary Figure 1.** (A) In P1, there is a significant difference in the distribution of fruit weight along the Y-axis; (B) In P3, there is a statistically significant difference in the distribution of fruit weight along the X-axis; (C) In P5, there is no statistically significant difference in the distribution of fruit weight along the X-axis.
